# Transcranial Direct Current Stimulation (tDCS) of the Right Inferior Frontal Gyrus Attenuates Skin Conductance Responses to Unpredictable Threat Conditions

**DOI:** 10.3389/fnhum.2016.00352

**Published:** 2016-07-12

**Authors:** Martin J. Herrmann, Jennifer S. Beier, Bibiane Simons, Thomas Polak

**Affiliations:** Laboratory for Psychophysiology and Functional Imaging, Department of Psychiatry, Psychosomatics and Psychotherapy, University Clinics of WürzburgWürzburg, Germany

**Keywords:** transcranial direct current stimulation, emotional regulation, sustained fear, right inferior frontal gyrus, NPU

## Abstract

Patients with panic and post-traumatic stress disorders seem to show increased psychophysiological reactions to conditions of unpredictable (U) threat, which has been discussed as a neurobiological marker of elevated levels of sustained fear in these disorders. Interestingly, a recent study found that the right inferior frontal gyrus (rIFG) is correlated to the successful regulation of sustained fear during U threat. Therefore this study aimed to examine the potential use of non-invasive brain stimulation to foster the rIFG by means of anodal transcranial direct current stimulation (tDCS) in order to reduce psychophysiological reactions to U threat. Twenty six participants were randomly assigned into an anodal and sham stimulation group in a double-blinded manner. Anodal and cathodal electrodes (7 * 5 cm) were positioned right frontal to target the rIFG. Stimulation intensity was *I* = 2 mA applied for 20 min during a task including U threat conditions (NPU-task). The effects of the NPU paradigm were measured by assessing the emotional startle modulation and the skin conductance response (SCR) at the outset of the different conditions. We found a significant interaction effect of condition × tDCS for the SCR (*F*_(2,48)_ = 6.3, *p* < 0.01) without main effects of condition and tDCS. *Post hoc* tests revealed that the increase in SCR from neutral (N) to U condition was significantly reduced in verum compared to the sham tDCS group (*t*_(24)_ = 3.84, *p* < 0.001). Our results emphasize the causal role of rIFG for emotional regulation and the potential use of tDCS to reduce apprehension during U threat conditions and therefore as a treatment for anxiety disorders.

## Introduction

Recently, it has been shown that patients with panic disorder (PD) were overly sensitive to unpredictable (U) threats Grillon et al. (2008) while showing a normal response to predictable (P; signaled) threats. Similar findings have been ascertained for posttraumatic stress disorder (PTSD), while generalized anxiety disorder (GAD) patients showed no difference compared to healthy controls (HCs; Grillon et al., 2009). For PD the anticipatory anxiety, and in consequence the sustained apprehension, is one key feature. Without any external cues, panic attacks are often perceived as U. For people with increased sensitivity to U conditions these situations might lead to sustained fear, which increases the probability of further panic attacks and might therefore contribute to the development of PD.

A common problem for anxiety patients is the difficulty to down-regulate negative emotions (Goldin et al., 2009), which in turn can lead to physical health concerns like cardiovascular disease (Suls and Bunde, 2005). An functional magnetic resonance imaging (fMRI) study with HCs showed that the instruction to down regulate their emotions during a paradigm of P and U threat lead to an activation of the rIFC (Klumpers et al., 2010), which was correlated with the success of down regulation.

This study confirmed recent studies which showed that the lateral prefrontal cortex (PFC) is generally activated during emotional regulation strategies such as reappraisal instruction (Ochsner et al., 2002; Ochsner and Gross, 2005; Eippert et al., 2007; Delgado et al., 2008). Evidence from functional imaging studies suggests that lateral PFC activation modulates the ventromedial PFC, which in turn inhibits amygdala responses (Ochsner et al., 2012). This supports a more general inhibitory function of the rIFC suggested by Aron et al. (2014). In their view, the rIFC, but not the dorsolateral PFC, is the locus of inhibition, which can be turned on in different modes. This motivates us to investigate whether we can booster the activation of the rIFC to enhance the spontaneous emotion regulation capacities during the the NPU-threat test, which allows the triggering of sustained fear (Schmitz and Grillon, 2012).

One strategy to modulate brain activation is the use of non-invasive brain stimulation methods. One of these non-invasive brain stimulation methods is transcranial direct current stimulation (tDCS), which has shown to modulate cognitive processes like selective attention (Stone and Tesche, 2009; Vierheilig et al., 2016) and working memory (Fregni et al., 2005), but also more emotional processes like error evaluation (Bellaïche et al., 2013) and fear memory consolidation (Asthana et al., 2013). Most relevant for our study, Jacobson et al. (2012) described that anodal tDCS to the right inferior frontal gyrus (rIFG) improves behavioral inhibition suggesting that tDCS modulates cognitive control in healthy individuals. Further studies add evidence to this assumption. For example Cunillera et al. (2014, 2015) repeatedly found higher inhibition (specifically proactive inhibition) induced by anodal tDCS on the rIFC by using a Go-NoGo and the stop-signal task (SST). Additionally Stramaccia et al. (2015) showed that the behavioral effects induced by rIFC tDCS (Jacobson et al., 2011; Ditye et al., 2012) are relatively long-lasting as measured in a SST 15 min after stimulation.

With regard to tDCS electrode positioning most studies (Jacobson et al., 2011, 2012; Cunillera et al., 2014, 2015; Stramaccia et al., 2015) used the crossing point between the T4-Fz and F8-Cz as position for the anodal electrode, although reference cathodal electrode positions varied throughout these studies. Furthermore, different electrode placements are used in various other experiments. Breitling et al. (2016) for example positioned the electrodes on F8 and posterior to the left mastoid when examining interference control effects of tDCS on adolescent attention deficit hyperactivity disorder (ADHD) patients. The results indicate better performance in the Flanker task for ADHD patients receiving anodal tDCS compared to the sham group in the first session. Another possible electrode position was used by Coffman et al. (2012), who positioned their anodal electrode near electrode site F10 to investigate the role of the rIFC in a target detection task.

In summary, these studies target the right IF-cortex or –gyrus when examining some kind of inhibition and position their anodal electrodes directly over the targeted area. In this study however, we used a neurotargeting software to determine the optimized stimulation montage. Many studies have revealed the importance of predictive modeling and the understanding of the complexity of current flow for precise targeting (Bikson et al., 2010; Datta et al., 2011; Dmochowski et al., 2011) compared to more intuitive positioning of electrodes “over” the targeted area. Thus, converging evidence points to tDCS as a successful tool to induce neuromodulatory effects and we therefore hypothesize that targeting the rIFC with anodal tDCS will reduce sustained fear induced by U threat.

## Materials and Methods

### Participants

Twenty six healthy participants were included in this study. After participants were given a complete description of the study and its procedures, written informed consent was obtained in accordance with the Declaration of Helsinki in its latest version from 2008. All procedures were approved by the Ethics Committee of the University Clinic of Würzburg. Participants were eligible for inclusion if they met the following criteria: (i) right handed; (ii) age 18–35 years. Participants were excluded from the trial if they met the following criteria: (i) any metal object or implant in brain, skull, scalp, or neck; (ii) implantable devices, including cardiac pacemakers and defibrillators; (iii) any neurological (like epilepsy or family history of epilepsy) or psychiatric illnesses; (iv) pregnancy; or (v) tinnitus. Information about these criteria was obtained by questionnaires. Participants were randomly assigned into a verum (*N* = 14) and a sham stimulation (*N* = 12) group.

To characterize the participants of this study and to ensure that the stimulation groups did not differ with respect to relevant anxiety traits, the anxiety sensitivity index (ASI3: Taylor et al., 2007), the positive and negative affect scale (PANAS: Watson et al., 1988), the spielberg state-trait anxiety inventory (STAI-T: Spielberger and Sydeman, 1994), the “Allgemeine Depressions Skala”, a screening instrument for depressive symptoms, in its short version (ADS-k: Hautzinger and Bailer, 1993), were completed by each participant (see Table 1).

**Table 1:** **Sample description**.

		Anodal (*N* = 14)		Sham (*N* = 12)		Statistics	
		M	SD	M	SD	*t*	*p*
**Age**		23.5	2.4	24.6	3.9	0.86	0.40
**STAI**	trait	35.9	6.5	37.9	9.1	0.67	0.51
	state t1	32.8	6.6	35.8	5.3	1.25	0.23
	t2	33.9	8.0	35.8	6.0	0.67	0.51
**ASI**	GAS	15.5	7.7	20.7	12.6	1.28	0.21
	BSM	3.9	4.1	5.8	5.6	1.00	0.33
	BSZ	7.6	3.1	9.4	4.8	1.13	0.27
	BKO	3.9	1.9	5.4	4.0	1.24	0.23
**ADS-K**		8.6	6.5	7.3	4.5	-0.55	0.59
**PANAS**	PA t1	31.1	6.3	30.9	6.2	-0.09	0.92
	t2	27.6	6.4	28.9	5.1	0.55	0.59
	NA t1	11.8	1.9	12.3	3.8	0.40	0.69
	t2	12.2	3.7	11.9	2.2	-0.25	0.81
**EHI**		0.9	0.1	0.9	0.1	−0.20	0.85

*Displayed are the means (M) and standard deviation (SD) for both stimulation groups and statistics for t-tests. ASI-GAS, anxiety sensitivity index 3 total; ASI-BSM, ASI subscore somatic; ASI-BSZ, ASI subscore social; ASU-BKO, ASI subscore cognitions; ADS-K, depression score; PANAS, positive and negative affect schedule before (t1) and after (t2) experiment; PA, positive affect; NA, negative affect; EHI, Edinburgh handedness inventory*.

### Paradigm

We used a classical NPU-task (Schmitz and Grillon, 2012), with a neutral (N), a P and an U condition. During U condition a blue square was presented on the screen with the information that an aversive scream can be presented at any time. During P condition a red circle was presented with the information that the scream was only presented during red circle presentation (Cue) but not during the interstimulus interval (ITI). During N condition a yellow triangle was presented together with the information that no scream will be presented at all. The paradigm was separated into seven blocks with two different sequences: (1) PNUNUNP; and (2) UNPNPNU. Each block was introduced by a 15 s instruction period. During each block, four cues were presented for 10 s, with an ITI varying between 11 and 22 s. After a block the instruction “Rest- please, relax” was displayed on the screen for 10 s. The aversive scream was presented two times for 2 s during each U and P block (sound #277, International Affective Digitized Sound, Center for Emotion and Attention University Florida, Bradley and Lang, 1999). Acoustic startle stimuli consisted of a 50 ms burst of white noise with 40 ms plateau and 5 ms rise and fall time delivered binaurally via in-ear headphones. No background sound was presented. Startle tones were presented three times during each block and ITI presentation, but not during rest after each block. Both sounds were delivered through an external sound card (Terratec, DMX 6 Fire USB) at an intensity of 103 db (sound pressure level, SPL). After data acquisition, participants rated all cues using a nine-point Likert scale (Self-Assessment Manikin Bradley and Lang, 1994 to assess arousal (1 = not arousing to 9 = very arousing).

### SCR and Startle Measurements

Skin conductance responses (SCRs) were recorded by a constant voltage circuit with 0.5 V across both electrodes and Brainamp ExG MR amplifier (Brain Products GmbH) with a sampling rate of 1000 Hz and a notch filter of 50 Hz. Responses were recorded via Vision Recorder Version (1.0) software (Brain Products GmbH) by using two Ag/AgCl electrodes (diameter = 13 mm) filled with non-hydrating gel. The two electrodes were attached to the thenar and hypothenar of the participants’ non-dominant palm. Raw data was offline low-pass filtered with 1 Hz, segmented and baseline-corrected 1000 ms prior to onset. SCR were characterized by peak responses in a time window of 1–5 s after onset of the cues. We analyzed only trials in which no startle tones were presented within the first 5 s. Artifact rejection was performed manually for every single trial. SCR values below 0.5 micro siemens were considered as null responses and coded as missing values. Participants with less than two valid trials in one of the conditions were excluded. SCR data were log-transformed (peaks +1) in order to account for inter-individual differences. Thereafter the values of each participant were *z*-transformed.

The eye blink component of the startle reflex was measured by recording electromyographic (EMG) activation of the right orbicularis oculi muscle (Brainamp ExG MR amplifier). Two 5 mm Ag/AgCl disc surface electrodes were positioned approximately 1 cm below the pupil and 1 cm below the lateral canthus of the right eye (impedance < 5 kΩ). The EMG-signal was filtered with a 28 Hz high-pass and a 499 Hz low-pass filter. A notch filter (50 Hz) was applied to control for components caused by (electro-)magnetic interference. After rectification signals were smoothed using a 50 ms moving average filter. Each segment was baseline-corrected 50 ms prior to the startle probe onset. Startle amplitudes were further defined as peak magnitudes (in microvolt) from the corrected EMG signal between 21 and 200 ms following probe onset. Artifact rejection was performed manually for every single peak. In order to allow for inter-individual differences, absolute blink magnitudes were normalized using T—standardization (Blumenthal et al., 2005). Participants with less than two valid trials in one of the conditions were excluded. Altogether, 24 participants had to be excluded due to missing valid data in SCR or startle data.

### DC Stimulation

tDCS was delivered by a battery-driven stimulator (DC-Stimulator-Plus, NeuroConn GmbH, Ilmenau, Germany) approved for use in humans. A pair of conductive-rubber electrodes (size 5 cm × 7 cm = 35 cm^2^) coated with Ten20 cream conductive paste (Waever and Company, Colorado, CO, USA) was positioned 1.5 cm posterior to EEG-position F8 (anodal) and 1.5 cm besides to EEG-position Fp1 (cathodal, in direction to Fpz). Electrode positions were chosen due to simulation with the neurotargeting software Soterix, indicating a direct current flow in the rIFG, (MNI position 52, 27, 14) with these electrode positions. As mentioned in the “Introduction” Section, the neurotargeting method has been shown to optimize electrode positioning by creating a direct current flow through the targeted area compared to simply positioning the electrode “over” the areal. But due to the large electrode sizes of 5 * 7 cm, this position also cover the electrode position previously used to target to rIFC (Jacobson et al., 2011, 2012; Cunillera et al., 2014, 2015; Stramaccia et al., 2015; Breitling et al., 2016). Stimulation intensity was *I* = 2 mA applied for 20 min parallel to the NPU task. The current was ramped up or down over the first and last 10 s of stimulation, respectively. The impedance was kept below 10 kΩ, controlled by the DC-stimulator. During sham condition the constant current was ramped up over the first 10 s once the DC had reached a current flow of 2 mA the current ramped down over 10 s. Therefore, the sham stimulation led to the same sensation in the participants, but had no long lasting effects (Iyer et al., 2005; Liebetanz et al., 2009).

### Statistics

Behavioral data was analyzed by repeated measures analysis of variance (ANOVA) and *t*-tests. For SCR analyses we calculated an ANOVA for repeated measurements with the factor condition (N P U) and the between-subject factor tDCS group (anodal vs. sham). Additionally we included the factor ITI (cue vs. no-cue presentation) for startle analyses. For all analyses the software SPSS (Version 23.0.0.0; SPSS, Inc.) was used. A probability level of *p* < 0.05 was considered statistically significant.

## Results

For SCR (see Table 2) we found a significant interaction effect for condition × tDCS (*F*_(2,48)_ = 6.3; *p* < 0.01), without a main effect for condition (*F*_(2,48)_ = 1.47; *p* = 0.24) or tDCS (*F*_(1,48)_ = 0.21; *p* = 0.65).

**Table 2:** **Mean and standard deviations (SDs) for the skin conductance response (SCR) amplitudes for both transcranial direct current stimulation (tDCS) groups in each NPU condition**.

	**Sham**		**Verum**		Statistics	
	M	SD	M	SD	*T*	*P*
**N**	−0.49	0.32	0.12	0.56	−3.30	0.003
**P**	0.08	0.60	0.16	0.54	−0.40	0.720
**U**	0.31	0.63	−0.34	0.46	3.00	0.006

**Figure 1:**
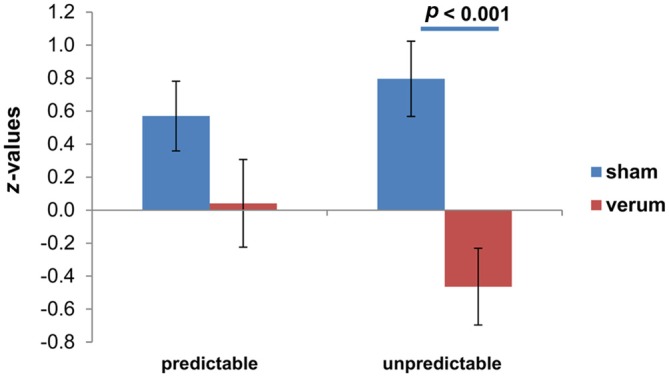
**Differential skin conductance response (SCR); (neutral (N)-[un]predictable) for both transcranial direct current stimulation (tDCS) groups.** Displayed are mean values with standard errors of the mean of *z*-standardized values.

The *post hoc* *t*-test revealed significant differences between sham and anodal group for the N and U conditions (see Table 2), leading to a significant lower increase from N to U condition in verum (*M* = −0.46 ± 0.23) compared to sham group (*M* = 0.80 ± 0.23; *t*_(24)_ = 3.84; *p* < 0.001; see Figure 1). The changes from N to P condition do not differ significantly between verum (*M* = 0.04 ± 0.27) and sham group (*M* = 0.57 ± 0.21; *t*_(24)_ = 1.52, *p* = 0.14).

For startle amplitudes (see Table 3) we found significant main effects for ITI (*F*_(2,24)_ = 53.6; *p* < 0.001), but no significant main effect for tDCS (*F*_(1,24)_ = 0.4; *p* = 0.84) or condition (*F*_(1,24)_ = 1.7; *p* = 0.19). The interactions condition × ITI (*F*_(2,48)_ = 7.9; *p* < 0.001) and tDCS × ITI (*F*_(2,48)_ = 4.4; *p* < 0.05) reached significance but not the interaction condition × ITI × tDCS (*F*_(2,48)_ = 0.6; *p* = 0.55).

**Table 3:** **Mean and SDs for the startle amplitudes for both tDCS groups in each NPU condition during cue presentation and interstimulus interval (ITI)**.

		**Sham**		**Verum**	
		M	SD	M	SD
**N**	Cue	46.5	2.96	45.7	2.61
	ITI	52.0	3.33	52.3	4.18
**P**	Cue	50.4	3.36	49.5	4.05
	ITI	50.5	3.42	52.7	2.63
**U**	Cue	47.4	4.87	45.1	4.68
	ITI	51.9	4.30	53.4	3.85

To analyze the interaction effect ITI × condition in more detail we calculated an ANOVA separately for the startle amplitudes during cue and ITI, with a significant main effect condition for startle amplitudes during cue presentation (*F*_(2,48)_ = 6.9; *p* < 0.01), but without effects during ITI presentation (*F*_(2,48)_ = 0.4; *p* = 0.65). *Post hoc* *t*-tests revealed that the amplitudes during cue presentation were significantly higher during P condition compared to N (*t*_(25)_ = 4.1; *p* < 0.001) and U (*t*_(25)_ = 2.8; *p* < 0.01) condition.

After the task the participants rated the cues of each condition for arousal. We found a main effect condition (*F*_(2,48)_ = 19.5; *p* < 0.001) without a main effect tDCS (*F*_(1,24)_ = 0.02; *p* = 0.90) or interaction effect condition × tDCS (*F*_(2,48)_ = 0.4; *p* = 0.69). *Post hoc* test revealed significant higher arousal ratings for P (*M* = 4.8; SD = 1.86; *t*_(25)_ = 5.5; *p* < 0.001) and U (*M* = 4.8; SD = 2.26; *t*_(25)_ = 5.2; *p* < 0.001) condition compared to N (*M* = 2.5; SD = 1.24).

## Discussion

In this pilot study we demonstrate that tDCS stimulation targeting the right IFG attenuates the psychophysiological reactions to cues indicating U threat. This result underscores the functional relevance of the right IFC for inhibitory processes in general (Aron et al., 2014; Cunillera et al., 2014), and especially for emotional regulation processes during sustained fear, as supposed by a previous fMRI study (Klumpers et al., 2010). This study (Klumpers et al., 2010) showed that the instruction to rest after each block leads to rIFC activation with a positive correlation between brain activation and fear reduction. Because activation of the rIFC was not found during the blocks, the authors interpreted the instruction to rest as a form of emotional regulation. Alternatively the instruction to rest might be a form of intentional inhibition of a motor response, which has shown to dampen the amygdala activation coincident with affective stimuli (Berkman et al., 2009).

In contrast to this interpretation, we found an effect of tDCS stimulation during the blocks of U threat. We therefore argue that our tDCS stimulation decreases apprehension without explicit emotional regulation instruction and should be further investigated as a potential tool to dampen stress responses.

Using a task with U durations of anticipation we found a phasic deactivation in the right IFC for the aversive compared to the N condition (Herrmann et al., 2016). This further supports the idea that this area is related to emotion regulation processes.

Unfortunately, these weakened responses during the blocks make it impossible to analyze the induced changes in SCR during rest in consequence of the emotion regulation instruction. In the verum tDCS group the SCR responses during the U threat block were low to the point that a further decrease during rest was not possible.

Beyond the reduced SCR to aversive stimuli we also found an effect for N cues with increased SCR in the verum stimulated group. In the sham stimulated group, we found a clear linear increase from N to P and U condition in SCR. In contrast to this, we found similar values for N and P condition, but clearly reduced SCR to U condition in our verum stimulated group. Therefore it is possible that in the verum stimulated group the differentiation between P and N was abolished for the sake of reduced arousal during U condition.

Interestingly, for startle amplitudes we only confirmed the effects of phasic fear (Grillon et al., 2008, 2009), with increased amplitudes during Cue presentation in P compared to the N and U condition. We could not show any sustained fear effects with increased startle amplitudes during ITI in the U compared to N condition. This might be an indication of reduced power in our sample, in which half of the participants were stimulated with tDCS.

One limitation of our study was that the startle burst presented during blocks prevented the analysis of the SCR for the whole block. During the verum tDCS the SCR was only weakened when the cues indicated U blocks, not during the complete block of sustained fear. A second pitfall in design was the high number of technical drop outs with the consequence of a small sample size. Further studies should use a modified design, for example with varying anticipation times (Herrmann et al., 2016) and without startle bursts, to extend the findings to a more sustained period of time. Another way to avoid these pitfalls is to choose a 2 × 2 design where participants complete the task under bother verum and sham condition. A further limitation of our tDCS study is that we can only calculate the current flow, but we cannot measure it. Indeed, we choose the electrode positions in our study based on previous studies and on software solution, but we cannot definitively confirm the exact current flow in the brain. Therefore, we can only suppose to have stimulated the rIFG.

Therefore this study should be understood as a starting point in a relatively new field of research with a lot of follow-up studies to further investigate and replicate the promising results obtained in our and other pioneer studies. In summary our study for the first time showed that tDCS as a form of non-invasive brain stimulation modulates U fear processing when targeted at the rIFC, which is highly relevant for the understanding and treatment of different anxiety disorders.

## Author Contributions

Conception of the work: MJH. Acquisition of the data: JSB. Analyses of the Data: MJH, JSB. Interpretation of data: TP, BS. Drafting the work or revising it critically for important intellectual content; MJH, JSB, BS, TP. Final approval of the version to be published; MJH, JSB, BS, TP. Agreement to be accountable for all aspects of the work: MJH, JSB, BS, TP.

## Funding

This study was funded by the Deutsche Forschungsgemeinschaft, SFB TRR 58, C06 and C07. The funding sources had no role in study design, data collection and analyses, decision to publish, or preparation of the manuscript. This publication was supported by the Open Access Publication Fund of the University of Wuerzburg.

## Conflict of Interest Statement

The authors declare that the research was conducted in the absence of any commercial or financial relationships that could be construed as a potential conflict of interest.
